# Functionally distinct contributions of the anterior and posterior putamen during sublexical and lexical reading

**DOI:** 10.3389/fnhum.2013.00787

**Published:** 2013-11-19

**Authors:** Marion Oberhuber, ‘Ōiwi Parker Jones, Thomas M. H. Hope, Susan Prejawa, Mohamed L. Seghier, David W. Green, Cathy J. Price

**Affiliations:** ^1^Wellcome Trust Centre for Neuroimaging, University College LondonLondon, UK; ^2^Wolfson College, University of OxfordOxford, UK; ^3^Cognitive, Perceptual and Brain Sciences, University College LondonLondon, UK

**Keywords:** fMRI, reading, word production, putamen, orthography, phonology

## Abstract

Previous studies have investigated orthographic-to-phonological mapping during reading by comparing brain activation for (1) reading words to object naming, or (2) reading pseudowords (e.g., “phume”) to words (e.g., “plume”). Here we combined both approaches to provide new insights into the underlying neural mechanisms. In fMRI data from 25 healthy adult readers, we first identified activation that was greater for reading words and pseudowords relative to picture and color naming. The most significant effect was observed in the left putamen, extending to both anterior and posterior borders. Second, consistent with previous studies, we show that both the anterior and posterior putamen are involved in articulating speech with greater activation during our overt speech production tasks (reading, repetition, object naming, and color naming) than silent one-back-matching on the same stimuli. Third, we compared putamen activation for words versus pseudowords during overt reading and auditory repetition. This revealed that the anterior putamen was most activated by reading pseudowords, whereas the posterior putamen was most activated by words irrespective of whether the task was reading words or auditory word repetition. The pseudoword effect in the anterior putamen is consistent with prior studies that associated this region with the initiation of novel sequences of movements. In contrast, the heightened word response in the posterior putamen is consistent with other studies that associated this region with “memory guided movement.” Our results illustrate how the functional dissociation between the anterior and posterior putamen supports sublexical and lexical processing during reading.

## INTRODUCTION

Reading involves the mapping of visual features (orthography) to meaning (semantics) and to articulatory codes (phonology) that will generate the corresponding speech sounds (phonetics). The non-semantic mapping from orthography-to-phonology can theoretically proceed lexically or sublexically (i.e., “champion” versus “cham”-“pi”-“on”), with sublexical processing enabling new or low frequency words (e.g., “jentacular”) to be read. The aim of our paper was to identify the brain areas associated with non-semantic orthographic-to-phonological mapping. We start by considering the cognitive processing that might be needed to support this function. We then review previous functional imaging approaches for identifying the associated brain regions, prior to introducing a novel experimental design that allows us to dissect different types of processing that explain the observed activation.

In cognitive terms, there are multiple levels at which orthography can be mapped to phonology within a single word. For example, in alphabetic scripts, phonology can be generated from a single letter (e.g., “s”); letter pair (e.g., “sh”), single syllables (e.g., “cham”), multi-syllables (e.g., “cham-pi”), and the whole word (e.g., “champion”). Critically, although the different levels are more or less consistent (e.g., the letter “m” has a similar sound by itself as in the word “champ”), there will also be multiple levels of inconsistencies, particularly in non-transparent languages like English (e.g., “c” has a different sound by itself than in “ch”). When lexical and sublexical outputs are consistent, sublexical processing can facilitate the production of the intended word (e.g., sublexical processing helps to distinguish the low-frequency word “animate” from the higher-frequency word “animal”), but when lexical and sublexical outputs are inconsistent, sublexical processing can interfere with word production, particularly for words with low lexical frequency (e.g., “yacht”). Accurate reading therefore requires the selection of articulatory codes that will support the intended pronunciation, and the inhibition of articulatory codes that are inconsistent with the intended pronunciation (e.g., “co-”, “count,” and “try” need to be suppressed when reading “country”). Finally, sublexical phonological codes need to be assembled in the right order with the correct prosody prior to speech production.

Previous functional neuroimaging approaches for identifying the brain regions associated with the non-semantic mapping of orthography to phonology have primarily involved the comparison of activation for reading pseudowords relative to reading familiar words (Binder et al., 2003; Jobard et al., 2003; Mechelli et al., 2003, 2005; Ischebeck et al., 2004; Dietz et al., 2005; Vigneau et al., 2005; Borowsky et al., 2006; Levy et al., 2008; Woollams et al., 2011). The rationale here is that pseudowords are more reliant on non-semantic orthographic to phonological mapping than words because the latter benefit from semantics. The trouble with this approach is that the higher activation for reading pseudowords than words could also arise because the visual inputs and articulatory sequences are less familiar. Therefore, more activation for pseudoword than word reading could reflect more “difficulty” at many levels of processing, not just the sublexical mapping of orthography-to-phonology.

An alternative approach is to include a further comparison in which we contrast reading aloud to picture naming (Bookheimer et al., 1995; Moore and Price, 1999; Price et al., 2006; Yoon et al., 2006; Borowsky et al., 2007; Mechelli et al., 2007; Seghier and Price, 2010; Kherif et al., 2011; Parker Jones et al., 2011; Vogel et al., 2013; Wheat et al., 2013). The rationale here is that word reading involves non-semantic mapping between visual inputs and phonology but object naming does not because (a) object parts provide semantic cues but not phonological cues to the object’s identity while (b) word parts (i.e., letters) provide the phonological cues but not semantic cues to the word’s identity. Unlike the comparison of reading pseudowords to reading words, the comparison of reading words to naming pictures can control for the demands on articulation and semantic content by using the same object names for both conditions (e.g., read the word “banana” versus name the picture of a banana). However, activation for reading relative to object naming does not control for the visual processing of orthographic inputs. Notably, the confounds associated with the contrast (reading words > picture naming) are different to those associated with (reading pseudowords > reading words). We can therefore minimize both sets of confounds by looking at what is commonly activated by (reading words > picture naming) and (reading pseudowords > words). This should isolate areas associated with the non-semantic mapping of orthography to phonology from (a) visual processing differences which are controlled in the comparison of pseudowords to words; and (b) general task demands/attention because reading is easier than object naming. To date, we are not aware of any neuroimaging study that has investigated such commonalities. We aimed to do so here.

The logic of our experimental design was as follows: to identify areas associated with non-semantic orthographic-to-phonological mapping, we compared activation for (reading words + reading pseudowords) to activation for (naming pictures of objects + naming the colors of meaningless, scrambled shapes). Activation that is higher for reading words than picture naming cannot be explained by word frequency differences or semantic content because the words were the written names of the same objects presented in the picture condition (i.e., they had the same semantics and word frequency). The influence of visual familiarity on our effects of interest was minimized because familiar and unfamiliar stimuli were balanced in the activation and baseline conditions (familiar words and unfamiliar pseudowords compared to familiar pictures of objects and unfamiliar pictures of scrambled objects). Any residual influence of visual familiarity could be tested by directly comparing the familiar to unfamiliar stimuli (i.e., familiar words and pictures of objects relative to unfamiliar pseudowords and scrambled objects).

Within the identified areas of interest, we compared activation for pseudowords and words. Our expectation was that pseudoword reading would increase the demands on sublexical processing because it is not supported by lexical or semantic processing. On the other hand, we hypothesized that greater activation for words than pseudowords might occur at the level of selecting articulatory codes from competing possibilities because of greater inconsistency between sublexical and lexical phonological codes (“country” versus “coun” and “try”) that will increase the demands on the suppression of mismatching codes.

**FIGURE 1:**
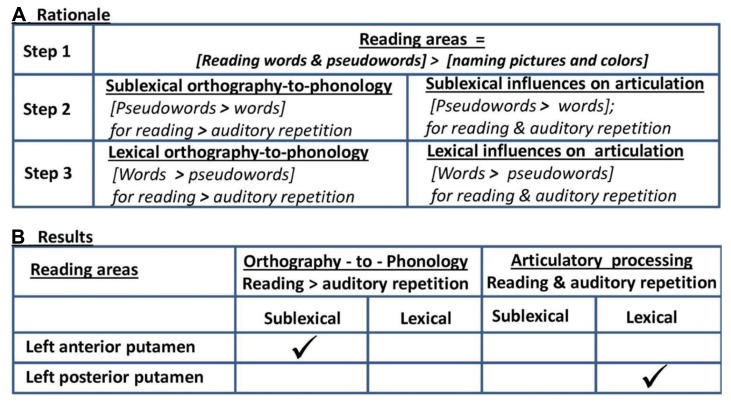
**Rationale and summary of results. (A)** Rationale: in Step 1, we identified areas that were involved in orthographic-to-phonological mapping as those that were more activated by reading words and pseudowords than naming objects in pictures and the color of scrambled pictures. Subsequent analyses were restricted to these regions of interest. In Step 2, we identified activation increases for sublexical reading where activation was greater for reading pseudowords than reading words. In Step 3, we identified activation increases for lexical reading where activation was greater for reading words than pseudowords. In Step 2 and Step 3, we distinguish activation that is specific to orthographic processing and activation arising at the level of articulation by testing whether the differences between pseudoword and word production was also observed during auditory repetition or not. **(B)** Results: the only area significant in Step 1 was the left putamen. The anterior putamen was more activated by reading pseudowords than any other condition, consistent with the influence of sublexical orthographic processing. The posterior putamen was more activated by words than pseudowords during reading and repetition, consistent with lexical influences at the level of articulation rather than orthography.

Our experimental design also included four auditory conditions that corresponded to the four visual conditions, namely (i) repetition of words, (ii) repetition of pseudowords, (iii) naming objects and animals from sounds (e.g., “cat” in response to “meow”), and (iv) naming the gender of a humming voice. This allowed us to isolate which of the areas that were more activated for reading than visual naming were also more activated by reading than auditory repetition. Greater activation for reading would indicate the influence of orthographic processing, whereas similar activation for auditory repetition and reading would indicate processing at the phonological/articulation level. More specifically, in areas activated by reading more than naming that were not activated by auditory repetition, we associated more activation for (i) pseudowords than words with sublexical orthographic-to-phonological conversion; and more activation for (ii) words compared to pseudowords with lexical influences on orthographic-to-phonological conversion. In contrast, in areas activated by reading more than naming that were also activated for auditory repetition, we associated more activation for (i) pseudowords than words with the demands on novel sequences of sublexical articulatory codes; and more activation for (ii) words compared to pseudowords with lexical influences on articulation (i.e., well-rehearsed motor outputs); see **Figure 1A**.

Finally, our experimental design was expanded to 16 conditions, by including a one-back-matching task for each of the eight types of stimuli (four visual, four auditory) used in the speech production tasks. The one-back-matching task involves viewing or listening to a series of stimuli and pressing a button when a stimulus is repeated. These conditions allowed us to identify (i) stimulus effects that were dependent or independent of task; (ii) areas that were involved in overt articulation, by comparing the speech production tasks to the one-back-matching tasks on the same stimuli. Effects of stimuli (e.g., pseudowords relative to words) that were independent of task must be arising at the subvocal level, whereas those that were greater for overt speech production are more likely to be related to articulation.

## MATERIALS AND METHODS

### SUBJECTS

Our sample initially included 26 healthy adults with no history of neurological conditions. One subject was subsequently excluded due to missing data in one of the conditions. The remaining 25 participants included 12 females and 13 males, aged 20–45 years (mean = 31.4, SD = 5.9 years). All were right handed (assessed with Edingburgh Handedness Inventory; Oldfield, 1971), native English speakers with normal or corrected-to-normal vision. They each gave written informed consent prior to the scanning and received financial compensation for their participation. The study has been approved by London Queen Square Research Ethics Committee (Study number NO32).

### EXPERIMENTAL DESIGN

The experiment comprised a 4 × 2 × 2 factorial design. Factor 1 compared stimuli with sublexical phonological properties (i.e., words and pseudowords) to stimuli without sublexical phonological properties (pictures of objects and meaningless shapes). Familiarity was controlled because half the stimuli were familiar (words, pictures of objects) and the others were unfamiliar (pseudowords, meaningless shapes). Factor 2 manipulated stimulus modality (visual or auditory). The four auditory stimuli were familiar words, unfamiliar pseudowords, environmental sounds associated with familiar objects or animals, and unfamiliar humming sounds. Factor 3 manipulated task (speech production or one-back-matching requiring a finger press response). The inclusion of the one-back-matching task allowed us to test whether activation (in areas activated by reading more than naming) was related to stimulus differences (e.g., written words versus pictures of objects) that were independent of task; or task effects (i.e., speech production versus one-back-matching) that were independent of stimulus.

### PARTICIPANT INSTRUCTIONS

In the speech production conditions, participants were instructed to (1) “Read words,” (2) “Read pseudowords” (3) “Name pictures of objects,” (4) “Name colors of meaningless shapes” (visual baseline condition), (5) “Repeat heard words,” (6) “Repeat heard pseudowords,” (7) “Name the source of environmental sounds” (i.e., CAMERA in response to the clicking noise of a camera), and (8) “Name the gender of a humming voice” (MALE or FEMALE; auditory baseline condition).

The one-back-matching task required a finger press response to indicate if the current stimulus was the same as the previous stimulus. To fully control for stimulus-effects, subjects were presented with exactly the same stimuli in both the speaking conditions and the one-back-matching conditions.

### STIMULUS SELECTION/CREATION

Stimulus selection started by generating 128 pictures of easily recognizable animals and objects (e.g., cow, bus, elephant, plate) with one to four syllables (mean = 1.59; SD = 0.73). Visual word stimuli were the written names of the 128 objects, with 3–12 letters (mean = 5 letters; SD = 1.8). Auditory word stimuli were the spoken names of the 128 objects (mean duration = 0.64 s; SD = 0.1), recorded by a native speaker of English with a Southern British accent approximating Received Pronunciation. Pseudowords were created using a non-word generator (Duyck et al., 2004) and matched to the real words for bigram frequency, number of orthographic neighbors, and word length. The same male speaker recorded the auditory words and pseudowords.

The non-verbal sounds associated with objects were available and easily recognizable for a quarter (32) of the stimuli, and taken from the NESSTI sound library (http://www.imaging.org.au/Nessti; Hocking et al., 2013). The duration of the environmental sounds needed to be significantly longer (mean length = 1.47 s, SD = 0.13) than the duration of the words [*t*(158) = 40.28; *p* < 0.001) because shorter sounds were not recognizable. The auditory baseline stimuli were recorded by both a male and female voice humming novel pseudowords, thereby removing any phonological or semantic content (mean duration = 1.04 s, SD = 0.43). Half of these stimuli were matched to the length of the auditory words (0.64); the other half, to the length of the environmental sounds (1.47). The visual baseline stimuli were meaningless object pictures, created by scrambling both global and local features, and then manually edited to accentuate one of 8 colors (brown, blue, orange, red, yellow, pink, purple, and green). Consistent speech production responses were ensured for all stimuli in a pilot study conducted on 19 participants.

### STIMULUS AND TASK COUNTERBALANCING

The 128 object stimuli were divided into four sets of 32 stimuli (A, B, C, and D). Set D was always presented as environmental non-verbal sounds. Sets A, B, and C were rotated across pictures, visual words, and auditory words in different participants. All items were therefore novel on first presentation of each stimulus type (for task 1); and the same items were repeated for task 2 but in a different condition. Half the subjects performed all eight speech production tasks first (task 1) followed by all eight one-back-matching tasks (task 2). The other half performed all eight one-back-matching tasks first (task 1) followed by all eight speech production tasks (task 2). Within each task, half the subjects were presented auditory stimuli first, followed by visual stimuli; and the other half were presented visual stimulus first followed by auditory stimuli. The order of the four stimulus types was fully counterbalanced across subjects, and full counterbalancing was achieved with 24 participants.

Each set of 32 items was split into 4 blocks of 8 stimuli, with one of the 8 stimuli repeated in each block to make a total of 9 stimuli per block (8 novel, one repeat). The stimulus repeat only needed to be detected and responded to (with a finger press) during the one-back-matching task.

### fMRI DATA ACQUISITION

Functional and anatomical data were collected on a 3-T scanner (Trio, Siemens, Erlangen, Germany) using a 12 channel head coil. To minimize movement during acquisition, a careful head fixation procedure was used when positioning each participant’s head. This ensured that none of the speech sessions were excluded after checking the realignment parameters. Functional images consisted of a gradient-echo EPI sequence and 3 × 3 mm in-plane resolution (TR/TE/flip angle = 3080 ms/30 ms/90°, EFOV = 192 mm, matrix size = 64 × 64, 44 slices, slice thickness = 2 mm, interslice gap = 1 mm, 62 image volumes per time series, including five “dummies” to allow for T1 equilibration effects). The TR was chosen to maximize whole brain coverage (44 slices) and to ensure that slice acquisition onset was offset synchronized with stimulus onset, which allowed for distributed sampling of slice acquisition across the study (Veltman et al., 2002).

For anatomical reference, a high-resolution T1 weighted structural image was acquired after completing the tasks using a three dimensional modified driven equilibrium Fourier transform (MDEFT) sequence (TR/TE/TI = 7.92/2.48/910 ms, flip angle = 16°, 176 slices, voxel size = 1 × 1 × 1 mm). The total scanning time was approximately 1 h and 20 min per subject, including set-up and the acquisition of the structural scan.

### PROCEDURE

Prior to scanning, each participant was trained on all tasks using different stimulus material, except for the environmental sounds which remained the same throughout both training and experiment. All speaking tasks required the subject to produce a single verbal response after each stimulus presentation. For the one-back-matching task, participants had to use two fingers of the same hand (right hand for half of the subjects, left hand for the other half) to press one of two buttons on a fMRI compatible button box to indicate whether the stimulus was the same as the one preceding it (left button for “same,” right button for “different”). The participants were instructed to keep their body and head as still as possible and to keep their eyes open throughout the experiment and attend to a fixation cross on the screen while listening to the auditory stimuli. Each of the 16 tasks was presented in a separate scan run, all of which were identical in structure. The script was written with COGENT and run in Matlab 2010a (Mathsworks, Sherbon, MA, USA).

Scanning started with the instructions “Get Ready” written on the in-scanner screen while five dummy scans were collected. This was followed by four blocks of stimuli (nine stimuli per block, 2.52 s inter-stimulus-interval, 16 s fixation between blocks, total run length = 3.2 min). Every stimulus block was preceded by a written instruction slide (e.g., “Repeat”), lasting 3.08 s each, which indicated the start of a new block and reminded subjects of the task. Visual stimuli were each displayed for 1.5 s. Each image was scaled to 350 × 350 pixels and subtended a visual angle of 7.4°, with a screen resolution of 1024 × 768. Words and pseudowords were presented in lower case Helvetica. Their visual angle ranged from 1.47 to 4.41° with the majority of words (with five letters) extending 1.84–2.2°.

The length of sound files varied across stimuli and tasks, ranging from 0.64 to 1.69 s (see stimulus creation above). Auditory stimuli were presented via MRI compatible headphones (MR Confon, Magdeburg, Germany), which filtered ambient in-scanner noise. Volume levels were adjusted for each subject before scanning. Each subject’s spoken responses were recorded via a noise-canceling MRI microphone (FOMRI IIITM Optoacoustics, Or-Yehuda, Israel), and transcribed manually for off-line analysis. We used eye-tracking to ensure participants were keeping their eyes open throughout the experiment.

### fMRI DATA PRE-PROCESSING

We performed fMRI data preprocessing and statistical analysis in SPM12 (Wellcome Trust Centre for Neuroimaging, London, UK), running on MATLAB 2012a (Mathsworks, Sherbon, MA, USA). Functional volumes were (a) spatially realigned to the first EPI volume and (b) un-warped to compensate for non-linear distortions caused by head movement or magnetic field inhomogeneity. We used the unwarping procedure in preference to including the realignment parameters as linear regressors in the first-level analysis because unwarping accounts for non-linear movement effects by modeling the interaction between movement and any inhomogeneity in the *T*2* signal. After realignment and unwarping, we checked the realignment parameters to ensure that participants moved less than one voxel (3 mm) movement within each scanning run. The anatomical T1 image was (c) co-registered to the mean EPI image which had been generated during the realignment step and then spatially normalized to the Montreal Neurological Institute (MNI) space using the new unified normalization-segmentation tool of SPM12. To spatially normalize all EPI scans to MNI space, (d) we applied the deformation field parameters that were obtained during the normalization of the anatomical T1 image. The original resolution of the different images was maintained during normalization (voxel size 1 mm × 1 mm × 1 mm for structural T1 and 3 mm × 3 mm × 3 mm for EPI images). After the normalization procedure, (e) functional images were spatially smoothed with a 6-mm full-width-half-maximum isotropic Gaussian kernel to compensate for residual anatomical variability and to permit application of Gaussian random-field theory for statistical inference (Friston et al., 1995).

### FIRST-LEVEL ANALYSES

In the first-level statistical analyses, each pre-processed functional volume was entered into a subject specific, fixed-effect analysis using the general linear model (Friston et al., 1995). All stimulus onset times were modeled as single events, with two regressors per run, one modeling instructions, and the other modeling the stimuli of interest. Stimulus functions were then convolved with a canonical hemodynamic response function. To exclude low-frequency confounds, the data were high-pass filtered using a set of discrete cosine basis functions with a cut-off period of 128 s. The contrasts of interest were generated for each of the 16 conditions (relative to fixation). The results of each individual were visually inspected to ensure that there were no visible artifacts (edge effects, activation in ventricles, etc.) that might have been caused by within scan head movements.

### EFFECTS OF INTEREST

At the second level, the 16 contrasts for each subject were entered into a within subjects one way ANOVA in SPM12. Statistical comparisons between different sets of conditions aimed to identify areas activated by reading more than naming and dissect these according to different levels of processing, as described below and illustrated in **Figure 1A**.

First we identified areas that were activated for reading words and pseudowords relative to picture and color naming (*p* < 0.05 FWE corrected for multiple comparisons across the whole brain). Second, within the identified areas of interest, we used an uncorrected statistical threshold to test whether activation was greater for pseudowords than words; distinguishing these areas as either activated for [pseudowords > words] during reading more than auditory repetition [i.e., the interaction of (pseudowords > words) and (reading > auditory repetition) or commonly activated for (pseudowords > words)] during both reading and auditory repetition (i.e., a main effect of pseudowords > words where there was no interaction with stimulus modality). Third, we repeated this process to test whether activation was greater for words than pseudowords; distinguishing (words > pseudowords) that was greater for reading than auditory repetition [i.e., the interaction of (words > pseudowords) and (reading > auditory repetition) or commonly activated for (words > pseudowords)] during both reading and auditory repetition (i.e., a main effect of words > pseudowords where there was no interaction with stimulus modality). The rationale for this three step approach is illustrated in **Figure 1A**. Fourth, in each region of interest, we examined the pattern of response across all 16 conditions to determine the type of processing that was being influenced by sublexical reading (e.g., articulation, visual processing).

### BEHAVIORAL ANALYSIS OF IN-SCANNER ACCURACY AND RESPONSE TIMES

Statistical analyses involved 2 × 4 ANOVAs in SPSS manipulating stimulus modality (visual versus auditory) with stimulus type (word, pseudoword, sound/picture, and gender/color). All ANOVAs were corrected for potential violations of sphericity, adjusting their degrees of freedom using the Greenhouse–Geisser correction (Greenhouse and Geisser, 1959). These corrections result in more conservative statistical tests (i.e., decreasing the risk of false positives while increasing the risk of false negatives), and account for the non-integer degrees of freedom below. Data from all 25 subjects were included for the speech production tasks (measuring accuracy in both visual and auditory modalities), while data from only 22 subjects were included for the one-back-matching tasks (measuring accuracy and response times (RTs) in both visual and auditory modalities). Three subjects’ data were excluded because their button press responses were not consistently detected (due to technical failure) in one of the following one-back-matching conditions (written pseudowords, environmental sounds, and spoken words).

## RESULTS

### fMRI RESULTS

#### Areas activated by reading more than naming

**FIGURE 2:**
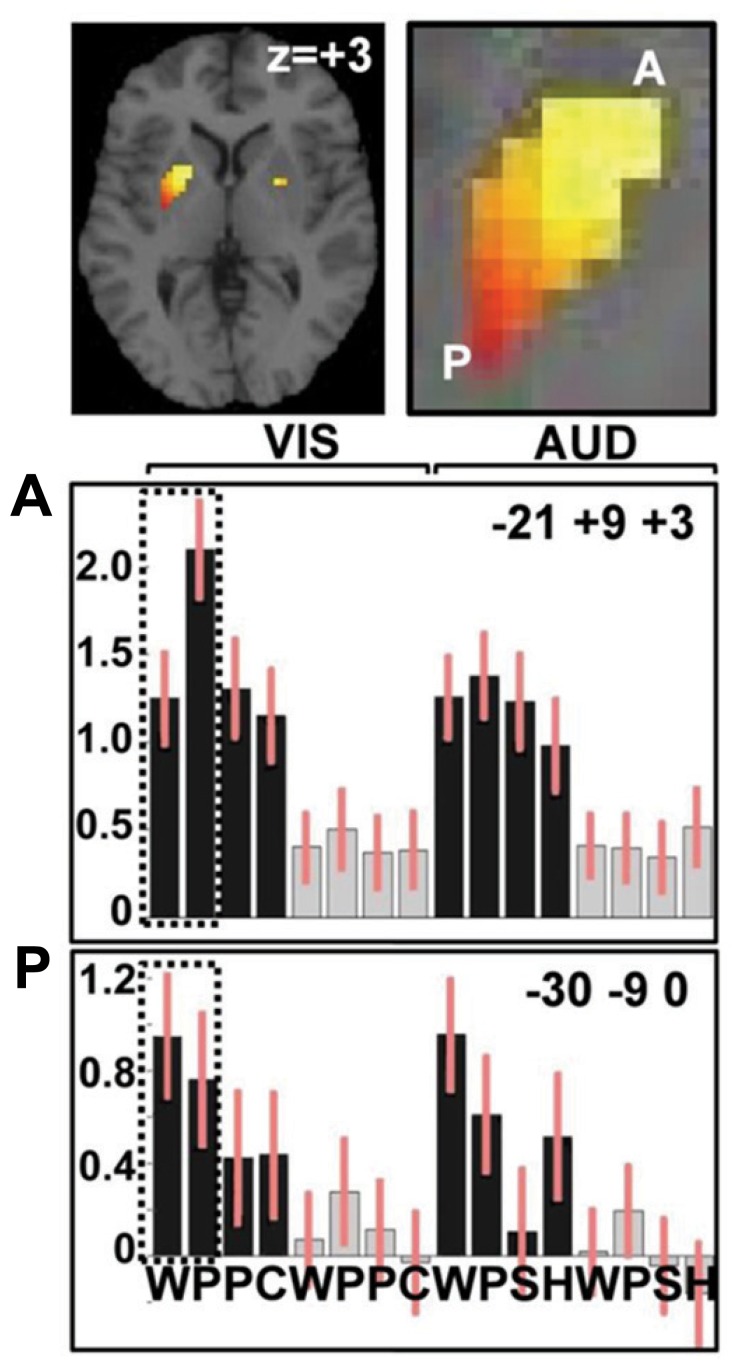
**Activation in the anterior and posterior putamen.** Top: activation in the putamen for reading words and pseudowords compared to naming objects and colors. A close up of the left putamen labels anterior putamen (A) and posterior putamen (P). Plots show relative activation for each of the 16 conditions in (A) at MNI co-ordinates *x* = -21, *y* = +9, *z* = +3 and (P) at MNI co-ordinates *x* = -30, *y* = -9, *z* = 0. Values on *y*-axis show activation relative to fixation (the baseline condition). Bars on each column indicate confidence intervals. Black represents speech tasks, gray represents one-back-matching task. The first eight columns represent the visual tasks with WPPC = word reading, pseudoword reading, picture naming, and color naming stimuli. The second eight columns represent the auditory tasks with WPSH = auditory repetition of words, auditory repetition of pseudowords, naming objects from non-verbal sounds and identifying gender of humming voice. Dotted frames highlight greater activation for pseudowords than words in anterior putamen and greater activation for words than pseudowords in posterior putamen. Details of the relevant statistics are provided in the text and **Table 1**.

Our areas of interest were defined as those that were more activated for reading words and pseudowords compared to object and color naming. Only one region reached a corrected level of significance (*p* < 0.05 FWE-corrected). This was a large area of the left putamen, reaching from the most anterior to the most posterior borders (see **Figure 2**; **Table 1**). The many other areas activated by either reading pseudowords relative to words; or reading words relative to picture naming are summarized below.

**Table 1:** Location and effects in the left and right putamen.

	Reading W&P > naming					P–W	
	Co-ordinates			Statistics		R–A	R&A
Location	*x*	*y*	*z*	*Z*sc	*k*	*Z*sc	*Z*sc
**L. Putamen**							
Anterior	–21	9	3	3.5	76	+3.3	ns
Peak	–24	3	3	4.7		ns	ns
Posterior	–30	–9	0	3.4		ns	*-*3.0
**R. Putamen**							
Anterior	24	6	6	3.2	21	+3.1	ns
Peak	24	0	9	3.9		ns	ns
Posterior	30	–9	9	3.2		ns	–1.6

*The location (in MNI space) and significance of the effects in the left and right putamen for reading words (W) and pseudowords (P) relative to picture naming and color naming. L = left hemisphere, R = right hemisphere. R – A = stimulus effects that are greater for reading > auditory repetition (i.e., an interaction of pseudowords versus words with stimulus modality). R&A = an effect of stimulus type that does not interact with stimulus modality (i.e., common to reading aloud and auditory repetition). *Z*sc = *Z* score, where negative *Z* scores indicate greater activation for words than pseudowords, and ns = not significant at *p* < 0.05 uncorrected. The co-ordinates at the peak and posterior putamen locations were identified in the main contrast (reading words and pseudowords relative to naming objects and colors). The peak *Z* score for the left putamen (highlighted in bold), and the number of voxels (k) that surpassed an uncorrected threshold of *p* < 0.001 were both significant after family wise error correction for multiple comparisons across the whole brain. In the right putamen, the corresponding *Z* scores and *K* are based on a statistical threshold of *p* < 0.001. The co-ordinates for the anterior putamen come from the direct comparison of pseudoword to word reading which was highly significant in the left hemisphere (*Z* score = 5.1, *p* < 0.05 after correction for multiple comparisons across the whole brain; *k* = 69 at *p* < 0.001 uncorrected) and significant at *p* < 0.001 at the corresponding location in the right hemisphere (*Z* score = 4.7; *k* = 29). The latter effects of stimuli in the reading task are not shown in the table that distinguishes the *Z* scores for the main effect of pseudowords relative words summed over the reading and auditory repetition conditions (R&A) from the interaction of stimuli and task (R – A) at the same voxel.*

#### Differential activation for pseudowords and words

Within the left putamen, greater activation for reading pseudowords than reading words was observed in the most anterior segment and an interaction between task and stimulus type indicated that the difference between pseudowords and words was greater during reading than during repetition (see **Table 1** for details). In contrast, greater activation for words than pseudowords was observed in the posterior putamen with no significant interaction between stimulus type (words versus pseudowords) and task (reading versus auditory repetition); see **Table 1**.

The contrasting responses to reading words and pseudowords in the anterior and posterior putamen was confirmed by a significant region by condition interaction [*F*(1, 24) = 25.4; *p* < 0.001, with Greenhouse–Geisser correction for non-sphericity), with greater activation for pseudoword than word reading in the anterior putamen but greater activation for word than pseudoword reading in the posterior putamen. This analysis was based on effect sizes from the peak voxel for pseudoword reading compared to word reading in the anterior putamen; and the peak voxel for word reading compared to pseudoword reading in the posterior putamen. The pattern of effects across all 16 conditions at these peak voxels is illustrated in **Figure 2**.

#### The response profile in the left anterior and posterior putamen across conditions

Although we found that activation in the left anterior putamen was more activated by reading pseudowords than any other condition (see **Figure 2**), activation was not specific to orthographic input. On the contrary, the left anterior putamen was activated by all speech conditions relative to one-back-matching on the same stimuli. We therefore describe the enhanced activation for pseudoword reading in the left anterior putamen as “the influence of sublexical orthographic processing on an articulation response.” Likewise, we observed that the increased activation for words over pseudowords in the left posterior putamen was not specific to reading, with comparable effects during auditory repetition (see **Figure 2**). An influence of sublexical phonology on the left posterior putamen was indicated by greater activation for words and pseudowords relative to picture and sound naming. Even higher activation for words than pseudowords indicates that left posterior putamen activation was most responsive when there was input from both lexical and sublexical phonology.

#### Response in anterior and posterior putamen in the right hemisphere

Our focus has been on the left putamen because this was the only area to be significantly more activated by reading words and pseudowords relative to naming pictures of objects and colors, when the statistical threshold was set at *p* < 0.05 FWE-corrected for multiple comparisons across the whole brain. However, *post hoc* analyses revealed that the pattern of effects observed in the left putamen were mirrored in the right putamen (see **Table 1**), albeit less significantly.

#### Greater activation for pseudowords than words, outside the putamen

Consistent with previous studies, we found many regions that were more activated for reading pseudowords than words, even though they were not more activated when pseudoword reading was compared to object naming. Greater activation for pseudowords than words that was common to reading and auditory repetition was observed in the left dorsal premotor cortex (MNI: -48, 0, +48), SMA/PreSMA (-6, +3, +66/0, +12, +51), bilateral posterior inferior frontal gyri (-45, +6, +24/+45, +9, +24), bilateral frontal operculum (-30, +21, 0/+33, +21, 0), left dorsal supramarginal gyrus (-42, -42, +45), and right cerebellum (+27, -63, -27). Greater activation for pseudowords than words that was dependent on task (reading > auditory repetition) was distributed bilaterally in occipital, occipito-temporal and the intraparietal cortices. All the above regions were identified after family wise error correction for multiple comparisons across the whole brain.

#### Greater activation for words than objects, outside the putamen

For completeness, we also looked for regions that were more activated for reading words than object naming even though they were not more activated for pseudoword relative to word reading. There was nothing significant in a whole brain search. Using regions of interest from Price et al. (2006), we found that reading relative to object naming that was common to the visual and auditory modalities (i.e., reading and repeating words relative to picture and sound naming), increased activation in the left premotor cortex (-54, -6, +33/-54, -6, +18) and the precuneus (-9, -53, +27/-3, -66, +36) with a non-significant trend in the left posterior superior temporal sulcus (-57, -42, +2). There were no regions that were more activated by words than object naming in the visual modality more than the auditory modality.

#### In-scanner behavior

**FIGURE 3:**
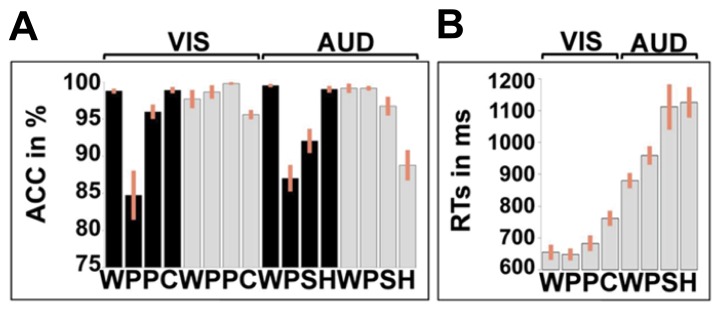
**In-scanner performance measures. (A)** Accuracy (ACC) in % correct for each of the 16 conditions, averaged over all 25 participants. **(B)** Response times (RTs) for the one-back-matching tasks for 22 participants (three subjects were excluded because of measurement error). Reaction times were not available for the speech production tasks. Unlike **Figure 2**, the bars show the standard error, not the confidence interval. As in **Figure 2**, black represents speech tasks, gray represents one-back-matching task. WPPC = word reading, pseudoword reading, picture naming, and color naming stimuli. The second eight columns represent the auditory tasks with WPSH = auditory repetition of words, auditory repetition of pseudowords, naming objects from non-verbal sounds and identifying gender of humming voice.

Details of the in-scanner speech production accuracy are provided in **Figure 3**. There was no significant effect of stimulus modality [*F*(1.00, 24.00) = 0.04; *p* = 0.84, Greenhouse–Geisser] but there was an effect of stimulus type [*F*(1.38, 33.11) = 29.14; *p* < 0.001, Greenhouse–Geisser) which interacted with stimulus modality [*F*(1.52, 36.41) = 3.82; *p* = 0.042, Greenhouse–Geisser). In the visual domain, accuracy was higher for words and colors than pictures and pseudowords. In the auditory domain, accuracy was higher for words and gender than sounds or pseudowords. RT data were not available for the speech production tasks. 

For accuracy in the one-back-matching task (with partially missing data for three subjects), we found a main effect of stimulus type [*F*(2.25, 47.32) = 29.94; *p* < 0.001, Greenhouse–Geisser], a main effect of stimulus modality [*F*(1.00, 21.00) = 4.89; *p* = 0.038, Greenhouse–Geisser] and a stimulus modality by condition interaction [*F*(2.08, 43.65) = 6.54; *p* = 0.003, Greenhouse–Geisser]. In the visual domain, accuracy was higher for pictures, pseudowords, and words relative to colors. Likewise, in the auditory domain, accuracy was higher for words, pseudowords, and sounds than gender. The lower accuracy for color and gender arose because some participants attempted to match these stimuli on their visual or auditory forms, rather than their color or pitch.

For RTs in the one-back-matching task, we found a main effect of stimulus type [*F*(1.62, 34.07) = 21.17; *p* < 0.001, Greenhouse–Geisser], a main effect of stimulus modality [*F*(1.00, 21.00) = 150.51; *p* < 0.001, Greenhouse–Geisser] and a stimulus modality by condition interaction [*F*(1.81, 38.00) = 6.68; *p* = 0.004, Greenhouse–Geisser]. For all conditions, participants were slower in the auditory modality than the visual modality. Within both stimulus modalities, RTs mirrored the accuracy on the one-back-matching task with faster RT and higher accuracy for words and pseudo-words compared to the baseline conditions (gender and color).

## DISCUSSION

Using a multi-factorial experimental design, we aimed to identify the brain areas where activation increases during the non-semantic mapping of orthography to phonology. Previous studies have addressed this question by looking at activation that is either greater for reading pseudowords than words or greater for word reading than object naming. In contrast, to avoid the confounds associated with each of these approaches, we identified areas associated with the non-semantic mapping of orthography to phonology as those where activation was greater for reading aloud words and pseudowords than for object or color naming. We also compared the effect of stimulus type during reading and auditory repetition to dissociate sublexical effects that were related to orthographic processing or articulation. Our logic was that effects that arose at the level of mapping orthography-to-phonology would be greater for reading than auditory repetition whereas effects that arose at the level of articulation would be common to reading and auditory repetition.

Our most significant finding was observed in the left putamen where we found that activation extending from the most anterior border to the most posterior border was greater for: (i) reading than picture naming; (ii) auditory repetition than sound naming; and (iii) producing speech than one-back-matching on the same stimuli. Within the putamen, the comparison of words to pseudowords revealed a striking and unexpected dissociation between the anterior and posterior territories. In the anterior putamen, activation was greater for reading pseudowords than any other condition, suggesting an influence of early orthographic processing on an articulatory area. In the posterior putamen, activation was greater for words than pseudowords during auditory repetition as well as during reading which demonstrates greater activation for more familiar speech output. The same pattern of response was observed in the right anterior and posterior putamen, albeit less significantly than in the left anterior and posterior putamen (see **Table 1**; **Figure 2**)

According to our task analysis (see **Figure 1**), increased demands on orthographic-to-phonological mapping will result in (i) greater activation for pseudowords than words, in areas (ii) more activated for words than object naming, but (iii) not more activated for auditory repetition of pseudowords than words. In contrast, greater activation for words than pseudowords was expected at the level of selecting articulatory codes from competing possibilities because words have greater inconsistency between sublexical and lexical phonological codes (“country” versus “coun” and “try”) that increases the demands on the suppression of mismatching codes. However, a role for the anterior putamen in orthographic-to-phonological mapping does not explain why the anterior putamen is also activated by object naming and other speech production tasks that do not involve orthographic-to-phonological mapping (see **Figure 2**). Moreover, a role for the posterior putamen in suppressing conflict between lexical and sublexical phonological codes does not explain why the posterior putamen is also more activated for words than pseudowords during auditory repetition (where there is no conflict between lexical and sublexical codes). We therefore turn to prior studies of the putamen to provide alternative interpretations of our findings.

Although we did not predict a double dissociation between the response of the anterior and posterior putamen to pseudowords and words, a *post hoc* literature search revealed that our findings are consistent with prior observations. For example, greater activation in the anterior putamen for reading pseudowords than words is consistent with prior studies that associated the anterior putamen with “the initiation of unskilled difficult movements” (Okuma and Yanagisawa, 2008; Aramaki et al., 2011), and the binding of sequential motor elements (Wymbs et al., 2012) as occurs during sublexical reading. Greater activation for pseudowords than words in the left anterior putamen during reading compared to auditory repetition can therefore be explained in terms of the demands on initiating or sequencing novel combinations of movements. Such demands will be less when the intended output is known (i.e., during auditory repetition) than when it can only be derived from visual cues (i.e., during reading). We are therefore not proposing that the anterior putamen is involved in orthographic-to-phonological mapping; instead, we are proposing that the output from orthographic-to-phonological mapping provides a more challenging trigger to the initiation of movements than the output of phonological processing during auditory repetition. Likewise, anterior putamen activation has been reported when the demands on articulation increase for non-native more than native language processing (Abutalebi et al., 2013) and late more than early bilingual speech (Frenck-Mestre et al., 2005). 

Plausibly, sub-articulatory processing might also explain why Kotz et al. (2002) found more activation in the anterior putamen when participants made lexical decisions on auditory words (confirming the stimuli were real words) relative to lexical decisions on pseudowords (rejecting stimuli as real words), while ignoring auditory primes, presented 100 ms before target onset, that induced different types of semantic interference for word targets than for pseudoword targets. Clearly, further investigation is required to confirm this and explain all the different effects that have been reported. Moreover, there may be multiple variables that influence activity in the anterior putamen. For example, the anterior putamen has been associated with eye movements (Petit et al., 2009; Neggers et al., 2012) which can explain our findings if we argue that eye movements increase when reading novel relative to familiar letter strings but does not readily explain greater activation for auditory words than pseudowords in Kotz et al. (2002). Note that Kotz et al. (2002) also report more activation for auditory pseudowords than auditory words in the “posterior putamen.” We do not discuss this result because the co-ordinates they report (Talairach: -32, +6, +9; MNI: -33, +9, +5) are far from those that we associate with the posterior putamen (MNI: -30, -9, 0).

With respect to our finding that activation in the posterior putamen was greater for words than pseudowords during auditory repetition and reading, we note that prior studies have associated the posterior putamen with “memory guided movement” (Menon et al., 2000; Tricomi et al., 2009). Activation in the posterior putamen may therefore be higher for word than pseudoword production because of familiarity with the required motor sequences. The preference of the posterior putamen for well-learnt movements and the anterior putamen for novel movements has been replicated in many other studies (Jueptner et al., 1997; Lehericy et al., 2005; Bapi et al., 2006; Fernandez-Seara et al., 2009) and concords with animal studies showing that injections of muscimol (GABA agonist) in the anterior putamen impairs learning of new sequences whereas injections into the middle-posterior putamen impairs the execution of well-learned sequences (Miyachi et al., 1997). More broadly, these differential responses in anterior and posterior putamen have been linked to the operation of different cortico-striatal loops. The anterior putamen interacts with anterior cortical areas (premotor cortex and anterior cingulate) as well as Broca’s area (Ford et al., 2013), whereas the posterior putamen interacts directly with the sensorimotor cortex, and cerebellum (Alexander et al., 1986; Jueptner et al., 1997; Hikosaka et al., 2002; Fernandez-Seara et al., 2009).

We have focused this paper on only one brain region (the putamen) because the pattern of response that we were looking for (greater activation for reading than picture naming; and reading pseudowords than words) did not reach significance in any other brain region. This was not due to a lack of sensitivity in all the other regions that have previously been associated with pseudoword reading more than word reading (Binder et al., 2003; Jobard et al., 2003; Mechelli et al., 2003, 2005; Ischebeck et al., 2004; Binder et al., 2005; Dietz et al., 2005; Vigneau et al., 2005; Borowsky et al., 2006; Levy et al., 2008; Woollams et al., 2011). On the contrary, we identified all the usual candidates for this contrast (pars opercularis, occipito-temporal gyrus, supramarginal gyrus etc.) but did not associate them with sublexical reading because they were not more activated for reading pseudowords than object naming. They may therefore represent levels of processing that are shared by pseudoword reading and object naming, such as enhanced demands on phonological retrieval and articulation relative to word reading. In addition, because our experimental design included both reading and auditory repetition tasks (see Hartwigsen et al., 2013), we are able to segregate areas that were more activated for pseudoword than word reading into those that were (i) also more activated by pseudowords than words during the auditory repetition task (i.e., at a post-orthographic level of processing), and those where the difference between pseudowords and words was greater for reading than auditory repetition (i.e., those related to orthographic processing). This revealed that all the anterior brain areas that were more activated for pseudoword than word reading (e.g., premotor, parietal, and SMA) were also more activated for auditory repetition of pseudowords than words. The commonality here is therefore assumed to arise in post-orthographic processing. In contrast, all the posterior brain areas that were more activated for reading pseudowords than words were not more activated by repetition of pseudowords than words. They are therefore likely to be associated with the visual processing that supports written word and object recognition.

To summarize, our findings are consistent with previous studies but offer a novel interpretation of activations that have previously been associated with pseudoword reading. By including multiple conditions we have dissociated the functions of the anterior and posterior putamen from areas that are involved in the visual processing that supports word and object recognition, or the articulatory processing that is common to reading and repetition.

## CONCLUSION

We have shown a functional dissociation between the anterior and posterior putamen. The response in the anterior putamen is consistent with prior studies that associated this region with “the initiation of unskilled difficult movements,” prior to motor output (Aramaki et al., 2011). In contrast, the response in the posterior putamen is consistent with prior studies that associated this region with “memory guided movement” (Tricomi et al., 2009). Prior studies have also noted a transition of activity from anterior to posterior putamen during visuo-motor sequence learning (Bapi et al., 2006). Here we show how the anterior and posterior putamen are differentially involved in lexical and sublexical reading.

## Conflict of Interest Statement

The authors declare that the research was conducted in the absence of any commercial or financial relationships that could be construed as a potential conflict of interest.

## AUTHOR CONTRIBUTIONS

Cathy J. Price, David W. Green, ‘Ōiwi Parker Jones, and Mohamed L. Seghier were responsible for the study design, Thomas M. H. Hope, ‘Ōiwi Parker Jones, and Susan Prejawa created the paradigm, Marion Oberhuber, Susan Prejawa, Thomas M. H. Hope, and ‘Ōiwi Parker Jones were involved in data acquisition and Marion Oberhuber and Susan Prejawa analyzed the data. Marion Oberhuber created the figures and searched the literature. All authors contributed to and approved the final manuscript.

## References

[B1] Abutalebi J. Rosa P. A. Castro Gonzaga A. K. Keim R. Costa A. Perani D. (2013). The role of the left putamen in multilingual language production. *Brain Lang.* 125 307–315 10.1016/j.bandl.2012.03.009 22538086

[B2] Alexander G. E. DeLong M. R. Strick P. L. (1986). Parallel organization of functionally segregated circuits linking basal ganglia and cortex. *Annu. Rev. Neurosci.* 9 357–381 10.1146/annurev.ne.09.030186.002041 3085570

[B3] Aramaki Y. Haruno M. Osu R. Sadato N. (2011). Movement initiation-locked activity of the anterior putamen predicts future movement instability in periodic bimanual movement. *J. Neurosci.* 31 9819–9823 10.1523/JNEUROSCI.4473-10.2011 21734273 PMC6703326

[B4] Bapi R. S. Miyapuram K. P. Graydon F. X. Doya K. (2006). fMRI investigation of cortical and subcortical networks in the learning of abstract and effector-specific representations of motor sequences. * Neuroimage* 32 714–727 10.1016/j.neuroimage.2006.04.205 16798015

[B5] Binder J. R. McKiernan K. A. Parsons M. E. Westbury C. F. Possing E. T. Kaufman J. N. (2003). Neural correlates of lexical access during visual word recognition. *J. Cogn. Neurosci.* 15 372–393 10.1162/089892903321593108 12729490

[B6] Binder J. R. Medler D. A. Desai R. Conant L. L. Liebenthal E. (2005). Some neurophysiological constraints on models of word naming. * Neuroimage* 27 677–693 10.1016/j.neuroimage.2005.04.029 15921937

[B7] Bookheimer S. Y. Zeffiro I. A. Blaxton T. Gaillard W. Theodore W. (1995). Regional cerebral blood flow during object naming and word reading. *Hum. Brain Mapp.* 3 93–106 10.1002/hbm.460030206

[B8] Borowsky R. Cummine J. Owen W. J. Friesen C. K. Shih F. Sarty G. E. (2006). FMRI of ventral and dorsal processing streams in basic reading processes: insular sensitivity to phonology. *Brain Topogr.* 18 233–239 10.1007/s10548-006-0001-2 16845597

[B9] Borowsky R. Esopenko C. Cummine J. Sarty G. E. (2007). Neural representations of visual words and objects: a functional MRI study on the modularity of reading and object processing. *Brain Topogr.* 20 89–96 10.1007/s10548-007-0034-1 17929158

[B10] Dietz N. A. Jones K. M. Gareau L. Zeffiro T. A. Eden G. F. (2005). Phonological decoding involves left posterior fusiform gyrus. *Hum. Brain Mapp.* 26 81–93 10.1002/hbm.20122 15934062 PMC6871728

[B11] Duyck W. Desmet T. Verbeke L. P. Brysbaert M. (2004). WordGen: a tool for word selection and nonword generation in Dutch, English, German, and French. *Behav. Res. Methods Instrum. Comput.* 36 488–499 10.3758/BF03195595 15641437

[B12] Fernandez-Seara M. A. Aznarez-Sanado M. Mengual E. Loayza F. R. Pastor M. A. (2009). Continuous performance of a novel motor sequence leads to highly correlated striatal and hippocampal perfusion increases. *Neuroimage* 47 1797–1808 10.1016/j.neuroimage.2009.05.061 19481611

[B13] Ford A. A. Triplett W. Sudhyadhom A. Gullett J. McGregor K. FitzGerald D. B. (2013). Broca’s area and its striatal and thalamic connections: a diffusion-MRI tractography study. *Front. Neuroanat.* 7:8 10.3389/fnana.2013.00008 PMC3650618 23675324

[B14] Frenck-Mestre C. Anton J. L. Roth M. Vaid J. Viallet F. (2005). Articulation in early and late bilinguals’ two languages: evidence from functional magnetic resonance imaging. *Neuroreport* 16 761–765 10.1097/00001756-200505120-00021 15858421

[B15] Friston K. J. Frith C. D. Turner R. Frackowiak R. S. (1995). Characterizing evoked hemodynamics with fMRI. *Neuroimage* 2 157–165 10.1006/nimg.1995.1018 9343598

[B16] Greenhouse S. W. Geisser S. (1959). On methods in the analysis of profile data. *Psychometrika* 24 95–112 10.1007/BF02289823

[B17] Hartwigsen G. Saur D. Price C. J. Baumgaertner A. Ulmer S. Siebner H. R. (2013). Increased facilitatory connectivity from the pre-SMA to the left dorsal premotor cortex during pseudoword repetition. *J. Cogn. Neurosci.* 25 580–594 10.1162/jocn_a_00342 23249347

[B18] Hikosaka O. Nakamura K. Sakai K. Nakahara H. (2002). Central mechanisms of motor skill learning. *Curr. Opin. Neurobiol.* 12 217–222 10.1016/S0959-4388(02)00307-0 12015240

[B19] Hocking J. Dzafic I. Kazovsky M. Copland D. A. (2013). NESSTI: norms for environmental sound stimuli. *PLoS ONE* 8:e73382. 10.1371/journal.pone.0073382 PMC3762767 24023866

[B20] Ischebeck A. Indefrey P. Usui N. Nose I. Hellwig F. Taira M. (2004). Reading in a regular orthography: an FMRI study investigating the role of visual familiarity. *J. Cogn. Neurosci.* 16 727–741 10.1162/089892904970708 15200701

[B21] Jobard G. Crivello F. Tzourio-Mazoyer N. (2003). Evaluation of the dual route theory of reading: a metanalysis of 35 neuroimaging studies. *Neuroimage* 20 693–712 10.1016/S1053-8119(03)00343-4 14568445

[B22] Jueptner M. Frith C. D. Brooks D. J. Frackowiak R. S. J. Passingham R. E. (1997). Anatomy of motor learning.2. Subcortical structures and learning by trial and error. *J. Neurophysiol.* 77 1325–1337 9084600 10.1152/jn.1997.77.3.1325

[B23] Kherif F. Josse G. Price C. J. (2011). Automatic top-down processing explains common left occipito-temporal responses to visual words and objects. *Cereb. Cortex* 21 103–114 10.1093/cercor/bhq063 20413450 PMC3000565

[B24] Kotz S. A. Cappa S. F. von Cramon D. Y. Friederici A. D. (2002). Modulation of the lexical-semantic network by auditory semantic priming: an event-related functional MRI study. *Neuroimage* 17 1761–1772 10.1006/nimg.2002.1316 12498750

[B25] Lehericy S. Benali H. Van de Moortele P. F. Pelegrini-Issac M. Waechter T. Ugurbil K. (2005). Distinct basal ganglia territories are engaged in early and advanced motor sequence learning. *Proc. Natl. Acad. Sci. U.S.A.* 102 12566–12571 10.1073/pnas.0502762102 16107540 PMC1194910

[B26] Levy J. Pernet C. Treserras S. Boulanouar K. Berry I. Aubry F. (2008). Piecemeal recruitment of left-lateralized brain areas during reading: a spatio-functional account. *Neuroimage* 43 581–591 10.1016/j.neuroimage.2008.08.008 18778780

[B27] Mechelli A. Crinion J. T. Long S. Friston K. J. Lambon Ralph M. A. Patterson K. (2005). Dissociating reading processes on the basis of neuronal interactions. *J. Cogn. Neurosci.* 17 1753–1765 10.1162/089892905774589190 16269111

[B28] Mechelli A. Gorno-Tempini M. L. Price C. J. (2003). Neuroimaging studies of word and pseudoword reading: consistencies, inconsistencies, and limitations. *J. Cogn. Neurosci.* 15 260–271 10.1162/089892903321208196 12676063

[B29] Mechelli A. Josephs O. Lambon Ralph M. A. McClelland J. L. Price C. J. (2007). Dissociating stimulus-driven semantic and phonological effect during reading and naming. *Hum. Brain Mapp.* 28 205–217 10.1002/hbm.20272 16767767 PMC3261378

[B30] Menon V. Anagnoson R. T. Glover G. H. Pfefferbaum A. (2000). Basal ganglia involvement in memory-guided movement sequencing. *Neuroreport* 11 3641–3645 10.1097/00001756-200011090-00048 11095535

[B31] Miyachi S. Hikosaka O. Miyashita K. Karadi Z. Rand M. K. (1997). Differential roles of monkey striatum in learning of sequential hand movement. *Exp. Brain Res.* 115 1–5 10.1007/PL00005669 9224828

[B32] Moore C. J. Price C. J. (1999). Three distinct ventral occipitotemporal regions for reading and object naming. *Neuroimage* 10 181–192 10.1006/nimg.1999.0450 10417250

[B33] Neggers S. F. Diepen R. M. Zandbelt B. B. Vink M. Mandl R. C. Gutteling T. P. (2012). A functional and structural investigation of the human fronto-basal volitional saccade network. *PLoS ONE* 7:e29517 10.1371/journal.pone.0029517 PMC3250458 22235303

[B34] Okuma Y. Yanagisawa N. (2008). The clinical spectrum of freezing of gait in Parkinson’s disease. *Mov. Disord.* 23 S426–S430 10.1002/mds.21934 18668623

[B35] Oldfield R. C. (1971). The assessment and analysis of handedness: the Edinburgh inventory. *Neuropsychologia* 9 97–113 10.1016/0028-3932(71)90067-4 5146491

[B36] Parker Jones O. Green D. W. Grogan A. Pliatsikas C. Filippopolitis K. Ali N. (2011). Where, when and why brain activation differs for bilinguals and monolinguals during picture naming and reading aloud. *Cereb. Cortex* 24 24 10.1093/cercor/bhr161 PMC3306575 21705392

[B37] Petit L. Zago L. Vigneau M. Andersson F. Crivello F. Mazoyer B. (2009). Functional asymmetries revealed in visually guided saccades: an FMRI study. *J. Neurophysiol.* 102 2994–3003 10.1152/jn.00280.2009 19710382

[B38] Price C. J. McCrory E. Noppeney U. Mechelli A. Moore C. J. Biggio N. (2006). How reading differs from object naming at the neuronal level. * Neuroimage* 29 643–648 10.1016/j.neuroimage.2005.07.044 16137894

[B39] Seghier M. L. Price C. J. (2010). Reading aloud boosts connectivity through the putamen. *Cereb. Cortex* 20 570–582 10.1093/cercor/bhp123 19561062 PMC2820698

[B40] Tricomi E. Balleine B. W O’Doherty J. P. (2009). A specific role for posterior dorsolateral striatum in human habit learning. *Eur. J. Neurosci.* 29 2225–2232 10.1111/j.1460-9568.2009.06796.x 19490086 PMC2758609

[B41] Veltman D. J. Mechelli A. Friston K. J. Price C. J. (2002). The importance of distributed sampling in blocked functional magnetic resonance imaging designs. *Neuroimage* 17 1203–1206 10.1006/nimg.2002.1242 12414260

[B42] Vigneau M. Jobard G. Mazoyer B. Tzourio-Mazoyer N. (2005). Word and non-word reading: what role for the visual word form area? *Neuroimage* 27 694–705 10.1016/j.neuroimage.2005.04.038 15961322

[B43] Vogel A. C. Church J. A. Power J. D. Miezin F. M. Petersen S. E. Schlaggar B. L. (2013). Functional network architecture of reading-related regions across development. *Brain Lang.* 125 231–243 10.1016/j.bandl.2012.12.016 23506969 PMC3863779

[B44] Wheat K. L. Cornelissen P. L. Sack A. T. Schuhmann T. Goebel R. Blomert L. (2013). Charting the functional relevance of Broca’s area for visual word recognition and picture naming in Dutch using fMRI-guided TMS. *Brain Lang.* 125 223–230 10.1016/j.bandl.2012.04.016 22632811

[B45] Woollams A. M. Silani G. Okada K. Patterson K. Price C. J. (2011). Word or word-like? Dissociating orthographic typicality from lexicality in the left occipito-temporal cortex. *J. Cogn. Neurosci.* 23 992–1002 10.1162/jocn.2010.21502 20429854 PMC4753674

[B46] Wymbs N. F. Bassett D. S. Mucha P. J. Porter M. A. Grafton S. T. (2012). Differential recruitment of the sensorimotor putamen and frontoparietal cortex during motor chunking in humans. *Neuron* 74 936–946 10.1016/j.neuron.2012.03.038 22681696 PMC3372854

[B47] Yoon H. W. Chung J. Y. Kim K. H. Song M. S. Park H. W. (2006). An fMRI study of Chinese character reading and picture naming by native Korean speakers. *Neurosci. Lett.* 392 90–95 10.1016/j.neulet.2005.09.027 16219423

